# Adsorption of Cr(VI) Using Organoclay/Alginate Hydrogel Beads and Their Application to Tannery Effluent

**DOI:** 10.3390/gels10120779

**Published:** 2024-11-28

**Authors:** Mayra X. Muñoz-Martinez, Iván F. Macías-Quiroga, Nancy R. Sanabria-González

**Affiliations:** 1 Departamento de Ingeniería Química, Universidad Nacional de Colombia Sede Manizales, Campus La Nubia, km 9 vía al Aeropuerto, Manizales 170003, Colombia; mxmunozm@unal.edu.co; 2 Departamento de Física y Química, Universidad Nacional de Colombia Sede Manizales, Campus La Nubia, km 9 vía al Aeropuerto, Manizales 170003, Colombia; ifmaciasq@unal.edu.co

**Keywords:** fixed-bed adsorption, Cr(VI), organobentonite, alginate, hydrogel, tannery wastewater, asymmetrical, fractal-like modified Thomas model

## Abstract

The tanning industry is among the most environmentally harmful activities globally due to the pollution of lakes and rivers from its effluents. Hexavalent chromium, a metal in tannery effluents, has adverse effects on human health and ecosystems, requiring the development of removal techniques. This study assessed the efficacy of organobentonite/alginate hydrogel beads in removing Cr(VI) from a fixed-bed adsorption column system. The synthesized organobentonite (OBent) was encapsulated in alginate, utilizing calcium chloride as a crosslinking agent to generate hydrogel beads. The effects of the volumetric flow rate, bed height, and initial Cr(VI) concentration on a synthetic sample were analyzed in the experiments in fixed-bed columns. The fractal-like modified Thomas model showed a good fit to the experimental data for the asymmetric breakthrough curves, confirmed by the high *R*^2^ correlation coefficients and low *χ*^2^ values. The application of organoclay/alginate hydrogel beads was confirmed with a wastewater sample from an artisanal tannery industry in Belén (Nariño, Colombia), in which a Cr(VI) removal greater than 99.81% was achieved. Organobentonite/alginate hydrogels offer the additional advantage of being composed of a biodegradable polymer (sodium alginate) and a natural material (bentonite-type clay), resulting in promising adsorbents for the removal of Cr(VI) from aqueous solutions in both synthetic and real water samples.

## 1. Introduction

Tannery industries have been recognized as the most polluting in the world due to the variety of chemical compounds that transform animal skins into leather. Tannery effluent frequently discharges chromium salts, phenolics, tannins, and other substances into the environment [1]. Using chromium sulfate as a tanning agent, the chrome tanning technique has been used in approximately 90% of tanneries worldwide [2]. This method offers significant advantages over vegetable tanning, including producing flexible, colorful, and versatile leather in a reduced time [2,3]. Approximately 40% of the chromium utilized in industrial tanneries is found in wastewater effluents, while artisanal tanneries do not eliminate this metal. Chromium in wastewater can exist as Cr(VI) and Cr(III), with the former being the more toxic form [2,4]. The severity of the pollution caused by tanneries is starkly illustrated by the chromium concentration in the wastewater of Bangladeshi tanneries, which ranges from 2656 to 5420 mg/L [5].

Although the tanning industry has modernized, artisanal leather production methods continue to be used in developing countries such as Colombia, which occupies the thirteenth place in leather manufacturing, with a share of 1.1% of world production. This industrial activity in Colombia is concentrated in the departments of Cundinamarca (81%) and Nariño (9.6%) [6]. Leather processing in the department of Nariño occurs mainly in the municipality of Belén, in small artisanal tanneries. While environmental authorities in Colombia have formulated regulations establishing the maximum permissible limit of total chromium in point discharges into surface water bodies and public sewage systems at 1.5 mg/L in this industry [7], this problem has not yet been mitigated.

The removal of pollutants in tannery wastewater, particularly Cr(VI), has been studied through different processes that include adsorption [8,9], reduction–adsorption [10], photodegradation [11], chemical reduction [12], membrane separation [13,14] and ion exchange [15]. Adsorption technology has attracted considerable interest among available treatments due to its simplicity, high efficiency, and scalability in various concentrations [16,17]. Various adsorbent materials that are able to remove Cr(VI) from water have been investigated, including clay-based materials [18], zeolites [19], zeolite/chitosan composites [20], layered double hydroxide–alginate microspheres [21], biochar [22], and activated carbon [23]. Clays and their modified derivatives are a group of adsorbent materials that can remove Cr(VI) from aqueous media [24,25]. Organoclays are synthesized by exchange with organic cations, generally quaternary ammonium salts [26,27], which improve the adsorption capacity for oxyanions of Cr(VI).

Alginate-based hydrogels have gathered particular attention in the past few years as functional materials for water treatment, specifically in applications for the adsorption of heavy metals and dyes [28,29]. Although alginate-base composites such as modified clay-enriched chitosan/alginate [30], alginate–montmorillonite/polyaniline [31], and calcium alginate–exfoliated clay [32] have been used for Cr(VI) adsorption, the previous investigations have used batch systems. This is the first study to use organoclay/alginate hydrogel beads to remove chromium oxyanions (HCrO_4_^−^ and Cr_2_O_7_^2−^) in a fixed-bed column, a small-scale representation of industrial continuous adsorption. The hydrogel beads were initially studied in batch adsorption tests for Cr(VI), analyzing the influences of the organoclay concentration in the hydrogel and the solution’s pH. Then, fixed-bed column experiments were carried out to evaluate the effects of the volumetric flow rate, bed height, and initial Cr(VI) concentration. Simple phenomenological models, such as Bohart–Adams, Thomas, and empirical Yoon–Nelson models, are often used to analyze breakthrough curves in column adsorption studies. These models represent symmetrical, S-shaped experimental breakthrough curves with a centrosymmetric point at 50% breakthrough [33]. In this study, the analysis of Cr(VI)’s dynamic adsorption was performed using the fractal-like modified Thomas model, which is capable of modeling asymmetric breakthrough curves. The efficacy of organoclay/alginate hydrogel beads was confirmed on a wastewater sample from an artisanal tannery industry.

## 2. Results and Discussion

### 2.1. Characterization of the Organobentonite/Sodium Alginate Beads

**Figure 1:**
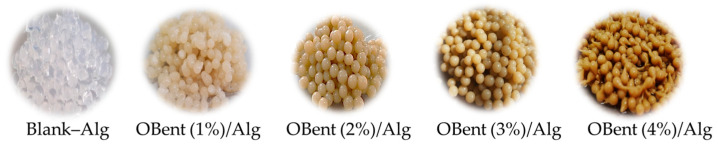
Color changes of the prepared hydrogel beads with different organoclay concentrations.

Figure 1 shows photographs of the prepared hydrogel beads with different concentrations of organobentonite, where Blank–Alg corresponds to the hydrogel alginate-only beads. As the concentration of OBent in the hydrogel increases, the color of the beads becomes browner. For a concentration of 4 wt.%, the beads exhibit a morphology with some irregularities.

**Figure 2:**
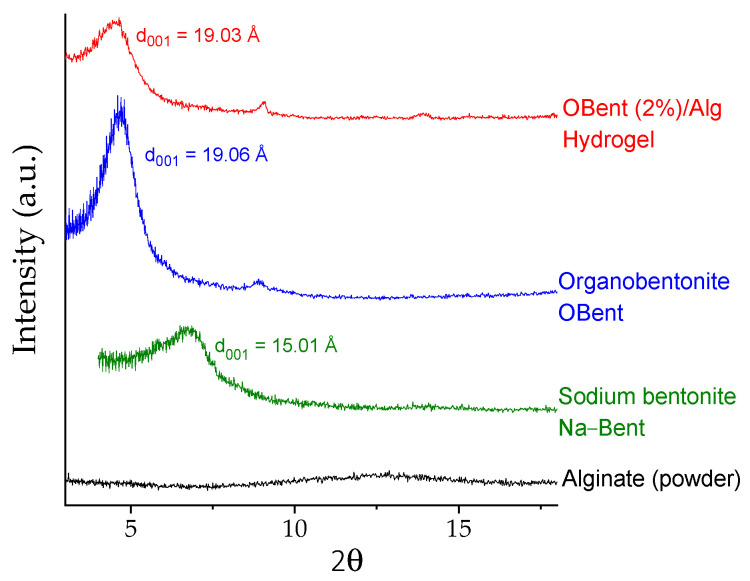
X-ray diffraction patterns of alginate, sodium bentonite, organobentonite, and the hydrogel beads with 2 wt.% organoclay.

Figure 2 illustrates the X-ray diffraction patterns of sodium bentonite (Na–Bent), organobentonite (OBent), alginate, and organoclay/alginate hydrogels, which are similar to those described in a previous article [16]. The Na–Bent diffractogram exhibited a d_001_ reflection of 15.01 Å, attributed to the smectite mineral in the clay [34]. The d_001_ value increased in the organoclay to 19.06 Å due to the intercalation of the hexadecyltrimethylammonium cation in the interlayer space of Na–Bent [16,27]. Alginate did not present X-ray diffraction signals and is considered an amorphous structure. To conclude, the X-ray diffraction pattern of OBent (2%)/Alg presented the characteristic signal of the organoclay, although with a decrease in the intensity of the signal, which was associated with the non-crystallinity of the alginate.

**Figure 3:**
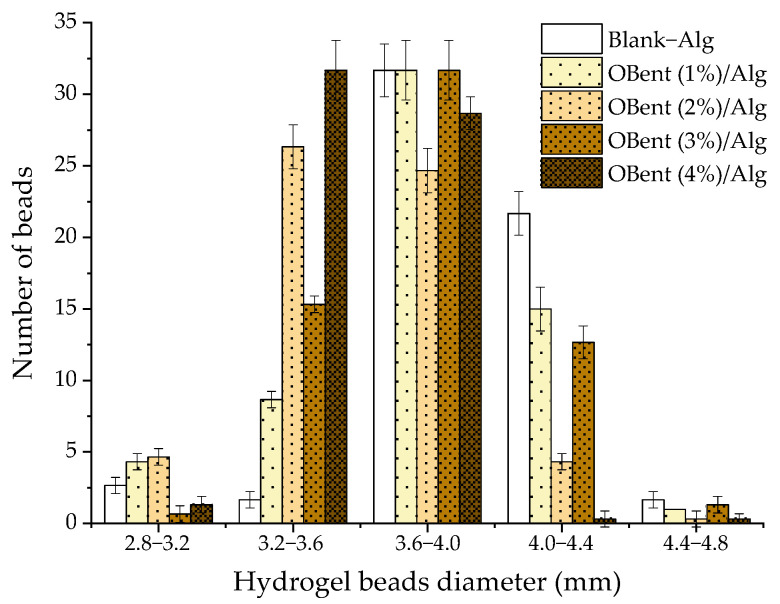
Size distribution (diameter) of the organobentonite/alginate hydrogel beads.

Figure 3 shows the size distribution of the hydrogel beads. The alginate or organobentonite/alginate mixture was dripped using the same Pasteur pipette to form the hydrogels. However, the size of the beads varied from 2.8 to 4.8 mm. Between 48 and 51% of the hydrogel beads exhibited a diameter ranging from 3.6 to 4.0 mm, regardless of the organoclay content.

The pH_ZPC_ of the OBent (2%)/Alg hydrogel beads was 7.3 ± 0.1, and at this pH value, the net surface adsorbent charge equaled zero. This parameter is essential to establish the affinity of the adsorbent material for a specific adsorbate, in this case, the Cr(VI) species. The HCrO_4_^−^ and Cr_2_O_7_^2−^ ions predominate in the pH range of 2–6 [35], and when the pH of the Cr(VI) solution < pH_ZPC_, the oxyanions can be electrostatically adsorbed by the organobentonite encapsulated in the alginate matrix.

### 2.2. Batch Cr(VI) Adsorption Tests Using the Organobentonite/Sodium Alginate Beads

**Figure 4:**
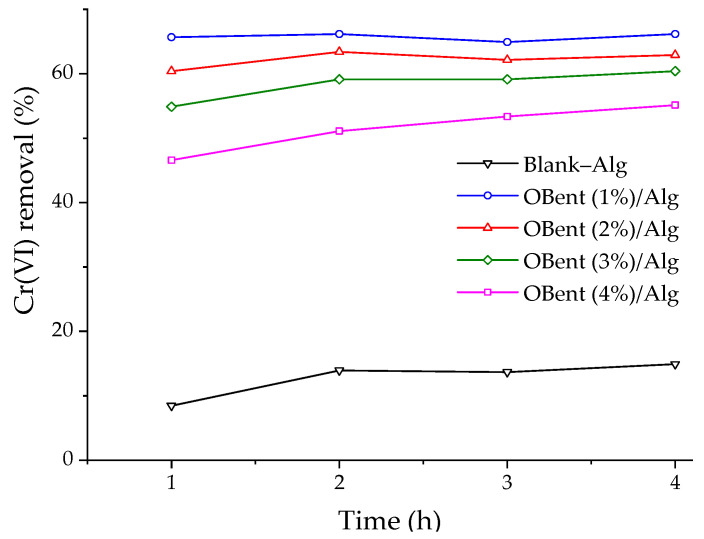
Effect of the organobentonite concentration in the hydrogel beads on Cr(VI) removal. Conditions: [Cr(VI)] = 25 mg/L, V = 50 mL, pH = 3.4, stirring speed = 150 rpm, organobentonite mass in hydrogel beads = 220 ± 3 mg, T = 20 ± 1 °C.

**Figure 5:**
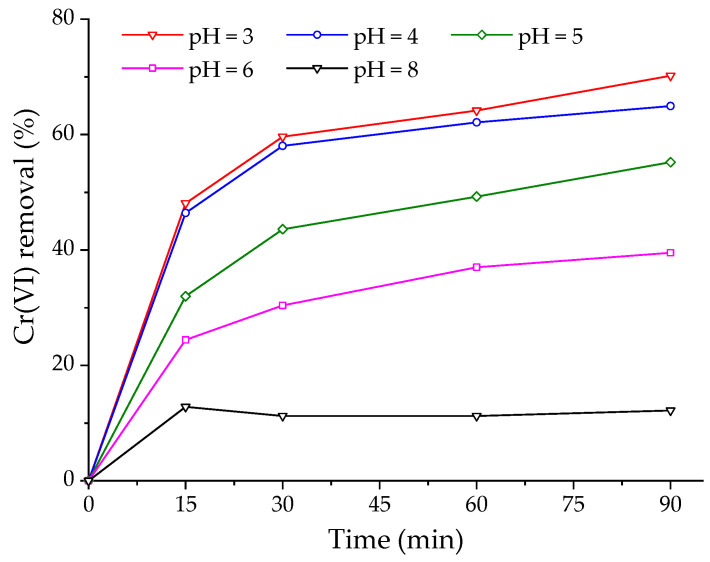
Effect of pH on Cr(VI) removal by OBent (2%)/Alg hydrogel beads. Conditions: [Cr(VI)] = 25 mg/L, V = 50 mL, stirring speed = 150 rpm, organobentonite mass in the hydrogel beads = 220 ± 3 mg, T = 20 ± 1 °C.

Batch experiments were performed to establish the particular operating conditions for the Cr(VI) adsorption column tests, examining the effects of the organobentonite content in the hydrogel beads (Figure 4) and the solution pH (Figure 5). The effect of the organoclay concentration in the beads was studied at the optimal pH value for Cr(VI) removal on HDTMA-modified Colombian bentonite described in a prior study [27]. As the concentration of organobentonite in the hydrogel beads increases, the removal efficiency of Cr(VI) decreases from 66.2% in OBent (1%)/Alg to 55.1% in OBent (4%)/Alg. Diffusional limitations can explain the previous result, as Cr(VI) species at a pH of 3.4 (HCrO_4_^−^ and Cr_2_O_7_^2−^) must cross the hydrogel surface to be electrostatically attracted by the positively charged cationic surfactant in the organobentonite [27]. A total of 14.9% of Cr(VI) was removed by alginate beads without organoclay (Blank–Alg), demonstrating that the organoclay had been the principal adsorbent.

Cr(VI) adsorption on OBent/Alg composites with 1, 2, and 3 wt.% organobentonite stabilized after the second hour of contact (Figure 4), indicating a rapid saturation of the active sites on the hydrogel beads [36]. Agrawal et al. [37] studied the adsorption of hexavalent chromium on alginate composites impregnated with iron oxide and found that adsorption occurs mainly during the first 30 min, and after this time, the removal tends to stabilize and equilibrium is reached. It is essential to mention that for the Cr(VI) removal tests with OBent (1%)/Alg and OBent (2%)/Alg, 296 and 159 beads were used, respectively, which guarantee a mass of 220 mg of organobentonite in the hydrogels. Although the OBent (1%)/Alg hydrogel beads had a higher Cr(VI) removal (66.2%), this was also due to the higher quantity of beads used. Therefore, it can be concluded that the OBent (2%)/Alg beads presented a better Cr(VI) adsorption capacity. Achazhiyath Edathil et al. [38] and Oussalah et al. [39] obtained similar results for the adsorption of organic acids and dyes (Congo red and methylene blue) using hybrid clay–alginate composites, where the amount of clay used for the preparation of composites was 2%.

Figure 5 shows the removal of Cr(VI) at different pH values as a function of time, which was favored at pH 3, with a maximum removal of 70.2% in 90 min. At pH 4, the removal of Cr(VI) decreased slightly to 64.9% at the same time, and for a pH higher than 4, a negative effect on removal was observed, with 12.2% at pH 8. This indicates that Cr(VI) adsorption decreases with increasing pH. Therefore, the removal efficiency of Cr(VI) depends on the pH of the solution and is controlled by the chromium species present at the different pH values and the charge of the adsorbent surface. HCrO_4_^−^ and Cr_2_O_7_^2−^ ions predominate in the pH range of 2–6, while CrO_4_^2−^ is the principal ion at pH > 6 [40,41].

From the above, it is concluded that at an acidic pH, mainly HCrO_4_^−^ ions are removed, which bind to the ammonium salt cation (HDTMA^+^) in the organobentonite encapsulated in alginate. At pH > 6, the removal of Cr(VI) decreases because a Cr_2_O_7_^2−^ ion requires two active sites of HDTMA^+^ for anion adsorption. This coincides with previous studies showing that the optimal pH for removing Cr(VI) with organoclay synthesized with HDTMA^+^ is 3.4 [27]. Therefore, a pH of 3.4 was selected for the following adsorption studies in a fixed-bed column.

### 2.3. Fixed-Bed Column Adsorption Tests for Cr(VI) Removal

**Figure 6:**
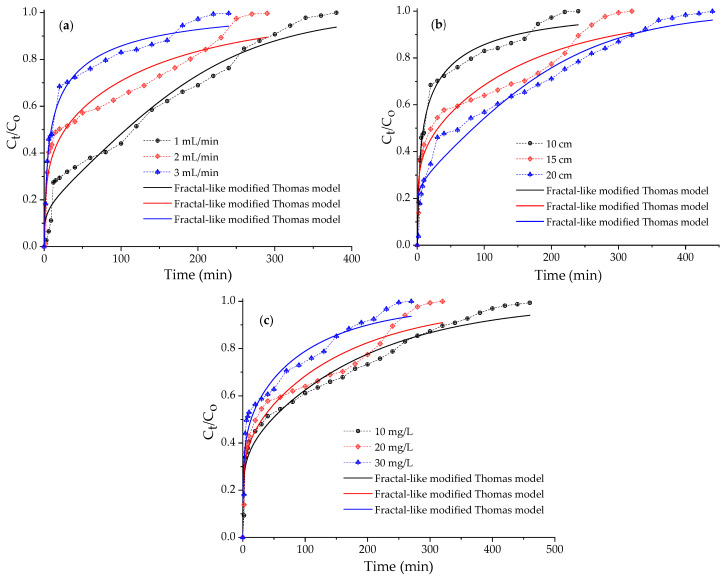
Breakthrough curves for Cr(VI) adsorption on OBent (2%)/Alg at different conditions (Dash line + symbol) and fits to the fractal-like modified Thomas model. (**a**) Effect of the volumetric flow rate, $C_{0}$ = 20 mg/L, pH = 3.4, and h = 10 cm; (**b**) effect of the bed height, $C_{0}$ = 20 mg/L, pH = 3.4, and Q = 3 mL/min; and (**c**) effect of the initial Cr(VI) concentration, pH = 3.4, Q = 3.0 mL/min, and h = 15 cm.

The effects of the volumetric flow rate, bed height, and initial Cr(VI) concentration were studied using fixed-bed columns with OBent (2%)/Alg hydrogel beads and a pH of the Cr(VI) aqueous solution of 3.4. Breakthrough curves for the different test conditions were obtained (Figure 6), and the adsorption parameters are summarized in Table 1.

By increasing the volumetric flow rate from 1 to 3 mL/min (Figure 6a), the breakthrough time ($t_{b}$) decreased from 8.25 to 1.09 min, as did the exhaustion time, ($t_{e}$), which was reduced from 323.50 to 183.36 min (Table 1). The adsorption capacity $q_{m}$ showed a slight decrease (3.1%) when the volumetric flow rate was increased from 1 to 3 mL/min. The effect of the flow rate on the column efficiency can be explained as a function of the contact time. As the volumetric flow rate increases, the flow rate of the Cr(VI) ions increases, leading to a lower resistance to mass transfer at the adsorbent surface and generating the rapid saturation of the column [42]. The contact time between the adsorbent and adsorbate is inversely related to the volumetric flow rate, and when Q (mL/min) increases, the Cr(VI) ions have a shorter contact time for diffusion and interaction with the active sites for adsorption [43].

Cr(VI) adsorption tests were performed on a column with 10, 15, and 20 cm of bed packed with OBent (2%)/Alg hydrogel beads to analyze the effect of the bed height on the breakthrough curves (Figure 6b). The breakthrough time ($t_{b}$) increased from 1.09 to 2.87 min as the bed height increased from 10 to 20 cm. The exhaustion time ($t_{e}$) was longer when the bed height was increased to 20 cm. The adsorption capacity ($q_{m}$) was increased by 51.7% when the bed height was doubled (from 10 to 20 cm). Therefore, a medium bed height, equivalent to 15 cm, was selected for the following tests. The increase in bed height favored Cr(VI) removal due to a greater amount of adsorbent in the column and greater availability of active sites for the adsorption of Cr(VI) ions [44]. In addition, the increase in bed height extended the contact time of the adsorbate ions with OBent (2%)/Alg hydrogel, favoring the adsorption process.

Column adsorption experiments varying the initial Cr(VI) concentration between 10 and 30 mg/L are shown in Figure 6c. By increasing the initial Cr(VI) concentration from 10 to 30 mg/L, the breakthrough time ($t_{b}$) and the exhaustion time ($t_{e}$) decreased, while the adsorption capacity ($q_{m}$) was favored, increasing by 64.0%. However, the Cr(VI) removal (*R*) decreased slightly (7.0%) with increasing adsorbate concentration. The increase in the adsorption capacity of Cr(VI) ions is attributed to a greater driving force provided by the rise in the initial concentration, lowering the resistance to mass transfer [44]. This behavior is similar to that reported by Nithya et al. [45], who performed adsorption tests of chromium ions in a column packed with acid-treated *Lantana camara* adsorbent.

It is important to highlight that for all continuous adsorption tests, Cr(VI) removal occurred in the first 20–30 min, and the shape of the breakthrough curve was asymmetric—with a rapid decrease in the concentration in the effluent, followed by a gradual approach to column saturation [46]. This behavior occurs when adsorption is diffusion-limited and occurs on heterogeneous surfaces [33,47]. Castro-Castro et al. [27] found that Cr(VI) adsorption on HDTMA-modified clay was very rapid and occurred in the first 20 min.

### 2.4. Breakthrough Curve Modelling for Cr(VI) Removal

Column adsorption studies frequently adopt classical breakthrough curve models with the S-shaped symmetrical sigmoid type at 50% breakthrough, where an irreversible equilibrium and simple reaction kinetics are assumed. The time-independent reaction rate is considered the sole curve-broadening factor that includes all other curve-broadening variables, accounting for all mass transport properties [33]. Since the breakthrough curve profiles for Cr(VI) adsorption illustrated in Figure 6 are asymmetric, the traditional Bohard–Adams and Thomas models do not fit the experimental data. This may be associated with high axial dispersion due to high flow rates or rapid adsorption [48]. For this reason, the breakthrough curves were fitted to the fractal-like modified Thomas model described in Table 2. The kinetic parameters of this model were determined by a nonlinear regression of the data using OriginLab^®^-Pro-8.1 software (OriginLab Corporation, Northampton, MA, USA).

Figure 6 shows the breakthrough curves’ experimental data and the fractal-like modified Thomas model fits at different volumetric flow rates, bed heights, and initial Cr(VI) concentrations. Table 3 presents the data for the constant of the fractal-like modified Thomas model ($K_{T,o}$) and the maximum adsorption capacity ($q_{o}$), together with the coefficients of determination ($R^{2}$) and the reduced chi-square value ($\chi^{2}$).

For the fractal-like modified Thomas model, as the flow rate increased from 1 to 3 mL/min, the $K_{T,o}$ value also increased (1.064 to 13.136 mL/mg min) owing to the diminished mass transport resistance with the increase in the volumetric flow rate [49,50]. The $K_{T,o}$ constant decreased as the height increased, and $q_{o}$ increased with the bed height. The behaviors of $K_{T,o}$ and $q_{o}$ with concentration were similar to that of the bed height. The Thomas constant typically increases with increasing Q values. It decreases with increasing h and $C_{o}$, indicating that the adsorption mechanism is not governed by mass transfer at the adsorbent interface [49,51]. As $C_{o}$ increased, the $K_{T,o}$ value decreased because the adsorption’s driving force was the difference in the Cr(VI) concentration between the adsorbent and solution. The behavior of the Thomas rate constant obtained in this research is similar to that of González-López et al. [52], who performed Cr(VI) adsorption tests in a packed bed column with chitosan composites.

The highest adsorption capacity ($q_{o}$ = 0.519 mg/g) achieved using the fractal-like modified Thomas model occurred at a bed height of 20 cm, with the lowest flow rate (1 mL/min) and an initial Cr(VI) concentration of 20 mg/L. The OBent/Alg hydrogel beads have approx. 2.0 wt.% OBent, and at a bed height of 10 cm (4.10 g), the active phase amounts to 0.082 g. The adsorption capacity of the organobentonite in the hydrogel is expressed using Equation (1):

(1) $$q_{o\;OBent}=0.519\;\frac{mg\;Cr(VI)}{g\;hydrogel}\times \left(\frac{4.100\;g\;hydrogel}{0.082\;g\;OBent}\right)=25.95\;\frac{mg\;Cr(VI)}{g\;OBent}$$

In general, the experimental data of the breakthrough curves fit well to the fractal-like modified Tomas model, with a high $R^{2}$ (close to 1.0) and low chi-square coefficient $\chi^{2}$ (close to zero) [16,53]. The value of the fractal component ($h_{f}$) is associated with the asymmetry of a diffusion-limited process. When the value of $h_{f}$ is close to zero, it indicates symmetry in the rupture curve, and the greater the deviation of $h_{f}$ from unity, the more pronounced the asymmetry [33]. The greatest asymmetry in the breakthrough curves was obtained for Q = 3 mL/min, h = 10 cm, and $C_{o}$ = 20 mg/L.

The maximum adsorption capacity of Cr(VI) on the organoclay/alginate hydrogel was compared with other composites, and the results are shown in Table 4. There are no adsorption studies of Cr(VI) on organoclay/alginate hydrogels in fixed-bed columns, and most of the research has focused on batch adsorption.

The $q_{m}$ value for Cr(VI) removal depends on the adsorbent material used (Table 4). Although, for this study, the maximum adsorption capacity of Cr(VI) obtained was 0.519 mg/g, it is essential to highlight that the hydrogel only contained 2 wt.% organobentonite; therefore, the $q_{m}$ value for the active phase in the hydrogel beads was 25.95 mg/g, a value similar to that obtained with alginate–goethite beads [54] and the crosslinked chitosan hydrogel functionalized with citric acid [56].

The capacity to regenerate and reuse the adsorbent is essential since disposing of it could negatively affect the environment and increase treatment costs [56]. Between the first and second cycles of adsorbent reuse, the OBent (2%)/Alg hydrogel beads showed a substantial decrease in the Cr(VI) desorption percentage, from 63.8 to 35.1%. The low desorption of Cr(VI) oxyanions could be associated with their strong interaction with the alkylammonium cations in the interlayer space of organoclay (active sites). Castro-Castro et al. studied the adsorption of Cr(VI) on organobentonite at 25 °C and determined the values of the equilibrium parameter $R_{L}$, a dimensionless constant obtained from the analysis of the Langmuir isotherm [27]. The $R_{L}$ values obtained ranged from 0.02 to 0.19, indicating that the adsorption is favorable (0 < $R_{L}$ < 1). However, $R_{L}$ values close to zero also suggest that adsorption is strong and tends to be irreversible ($R_{L}$ = 0) [59].

### 2.5. Removal of Cr(VI) from a Sample of Tannery Wastewater

Table 5 summarizes the results of the physicochemical characterization of the effluent from an artisanal tannery in Belén (Nariño, Colombia). Typical tannery wastewater has a very high chemical oxygen demand (COD) and inorganic contaminants, such as chromium, at concentrations ranging from 10 to 1000 mg/L [60] and exceeding the discharge limits established in the legislation in Colombia [7]. The conductivity and Cr(VI) concentration parameters do not have an established limit but must be analyzed and reported.

**Figure 7:**
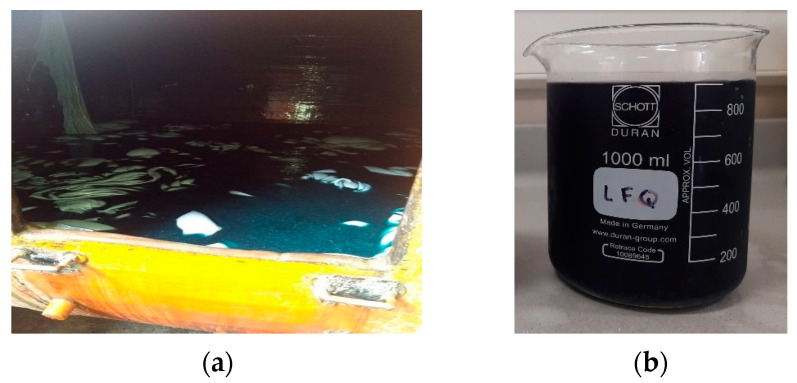
(**a**) Leather tanning tank; (**b**) sample of tannery wastewater.

Figure 7 shows a photograph of the leather tanning tank (Figure 7a) and a sample from the artisanal tannery wastewater (Figure 7b). The wastewater is dark blue, attributed to the chromium sulfate salts used in tanning leather. Although this research focuses on the removal of Cr(VI), given the high concentration of Cr(III) in the tannery wastewater, before the Cr(VI) adsorption tests in the fixed-bed column, 99.82% of the Cr(III) was removed in a column packed with 2 wt.% sodium bentonite hydrogel beads (results not included in this publication), leaving the effluent with 4.3 ± 0.5 mg/L of Cr(III). Trivalent chromium was removed by exchanging Cr^3+^ cations for sodium ions (Na^+^) in the interlaminar space of the sodium bentonite [61].

**Table 5:** Physicochemical characteristics of tannery wastewater.

Parameter	Standard Method	TWW	Typical Composition [62,63,64]	Permissible Limits [7]
pH	4500-H^+^ B	4.1 ± 0.3	8–11	6.0–9.0
Conductivity (μS/cm)	2510 B	16,191 ± 318	6300–9100	Not established
COD, mg/L	5220 D	2352 ± 269	9922–10,180	1200
Sulfates, mg/L	4500-${SO}_{4}^{2–}$ E	9497 ± 635	1951–574	Not established
Total chromium, mg/L	3111 B	2515 ± 118	60–100	1.5
Cr(III), mg/L	3111 B	2359 ± 108		Not established
Cr(VI), mg/L	3500-Cr B	156 ± 7	<0.11	Not established

**Figure 8:**
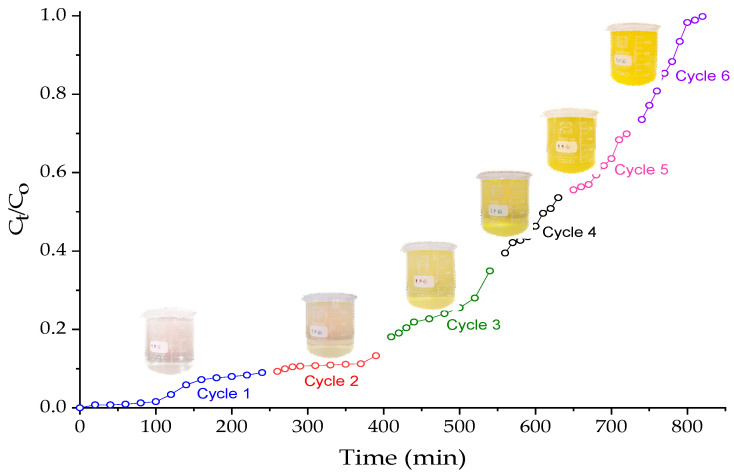
Successive Cr(VI) adsorption cycles on OBent (2%)/Alg hydrogel beads.

After Cr(III) adsorption, the process effluent was passed through a packed bed column with OBent (2%)/Alg hydrogel beads under the following experimental conditions: sample volume = 1000 mL, solution pH = 3.4, amount of adsorbent in the column = 143.94 g, flow rate = 7.0 mL/min, and bed height = 25 cm. To achieve column saturation, six adsorption cycles were performed with 1 L of tannery wastewater each (Figure 8), going from 156 ± 17 mg/L of Cr(IV) to an effluent with 0.3 ± 0.1 mg/L of Cr(VI), equivalent to the removal of 99.81% of Cr(VI).

**Figure 9:**
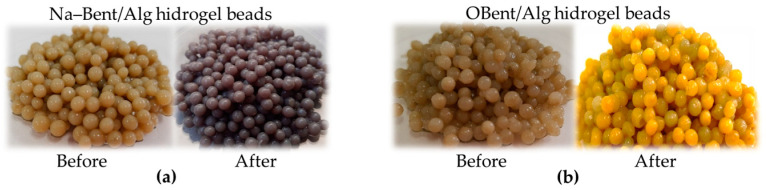
Appearance of hydrogel beads. (**a**) Before and after Cr(III) removal; (**b**) before and after Cr(VI) removal.

Figure 9 shows the hydrogel before and after the removal of Cr(III) and Cr(VI). The Na–Bent (2%)/Alg hydrogel beads changed from a light brown color (typical of bentonite) to blue after the removal of Cr(III), the characteristic color of a chromium sulfate solution, Cr_2_(SO_4_)_3_. The OBent (2%)/Alg hydrogel beads turned from a light brown color to a yellow color after six cycles of Cr(VI) adsorption, and this is typical of chromate ions (CrO_4_^2–^) in solution.

## 3. Conclusions

In this research, the encapsulation of organobentonite in a sodium alginate matrix was carried out, leading to the formation of organoclay/alginate hydrogel beads (OBent/Alg). Through an XRD analysis of the hydrogel beads, it was confirmed that the structure of the organobentonite was preserved after its encapsulation in the alginate matrix.

Based on batch Cr(VI) adsorption tests, it was found that the concentration of organoclay in the hydrogel had an inverse effect on the removal of this metal. This can be attributed to a high concentration of organoclay, which generates diffusional limitations, decreasing the adsorption capacity of the active sites in the adsorbent material. The most efficient hydrogel beads for Cr(VI) removal were those with 1.0 and 2.0 wt.% organobentonite.

The shape of the breakthrough curves for removal of Cr(VI) by OBent (2%)/Alg hydrogel beads was asymmetric, and this behavior occurs when adsorption is diffusion-limited. The adsorption of Cr(VI) was favored by a decrease in the volumetric flow rate and an increase in the bed height and the initial adsorbate concentration due to a longer contact time, increased adsorbent amounts, and a higher concentration gradient.

The maximum adsorption capacity of Cr(VI) obtained in the column tests with the fractal-like modified Thomas model was 0.519 mg/g hydrogel under the following conditions: flow rate of 1 mL/min, bed height of 10 cm, and initial concentration of 20 mg/L. Between the first and second cycles of adsorbent reuse, the Cr(VI) desorption percentage on OBent (2%)/Alg hydrogel beads decreased drastically from 63.8 to 35.1%.

The tannery wastewater sample had a high chromium content. The adsorption tests on the column showed excellent results, obtaining a Cr(VI) removal rate of 99.81%. Organobentonite/alginate hydrogel beads are promising adsorbents for the removal of chromium from aqueous solutions, with the added advantage of being prepared from a low-cost natural material (bentonite-type clay) and a biodegradable polymer (sodium alginate).

## 4. Materials and Methods

### 4.1. Reagents and Materials

Initial Cr(VI) adsorption experiments were conducted using a synthetic water sample. A stock solution of 1000 mg/L Cr(VI) was obtained by dissolving 0.1 N K_2_Cr_2_O_7_ (Titrisol, Merck KGaA, Darmstadt, Germany) in deionized water, which was subsequently used for solution preparation by dilution. Sodium alginate (C_6_H_9_NaO_7_, purity > 91%, Loba Chemie Pvt. Ltd., Mumbai, India) and the surfactant hexadecyltrimethylammonium bromide (HDTMA–Br, purity > 98.0%, PanReac AppliChem, Barcelona, Spain) were of analytical grade.

### 4.2. Preparation of the Organobentonite/Sodium Alginate Beads

Purified bentonite (Bent), with a previously published chemical and mineralogical composition [34], was used to synthesize organoclay (OBent). The intercalation of clay with hexadecyltrimethylammonium bromide (HDTMA–Br) was performed according to the methodology established by the research group [16,65]. A quantity of surfactant equal to 1.5 times the cation exchange capacity of the clay (63.02 meq/100 g) was selected for the modification. Rivera-Arenas et al. [16] previously published the details of the synthesis process of organoclay/alginate hydrogels and their characterization. The procedure involved the combination of a defined quantity of organoclay (OBent) with an aqueous sodium alginate solution (Na–Alg, 1 g/100 mL, 60 °C, 1 h at 250 rpm) to obtain final suspension concentrations of 1, 2, 3, and 4 wt.%. The suspension was gradually introduced into a 4 wt.% CaCl_2_ solution at 20 ± 1 °C using a Pasteur pipette, following 1 h of mixing the organoclay and Na–Alg solution. The hydrogel beads were immersed in a CaCl_2_ solution for three hours to ensure Ca^2+^ crosslinking. OBent (wt.%)/Alg was a label assigned to the hydrogel beads, where wt.% denotes the mass percentage of OBent used in the synthesis. The hydrogel beads made entirely of alginate were designated as Blank–Alg.

The hydrogel beads (OBent (2%)/Alg) and the raw materials used for their synthesis (sodium bentonite, organobentonite, and alginate) were characterized by X-ray diffraction (LabX Shimadzu XRD-6000 diffractometer, Kyoto, Japan). The average diameters and size distribution of the hydrogel beads were determined from photographs analyzed with ImageJ3 software, developed by the National Institute of Health (Bethesda, MD, USA). The diameter of 60 hydrogel beads was measured in each photograph, and the bead size distribution corresponded to the measurements of the three samples. The point of zero charge (pH_PZC_) of the OBent (2%)/Alg hydrogel beads was determined using the salt addition method [66].

### 4.3. Batch Adsorption Tests

The operating conditions for the column tests were established using batch adsorption studies of Cr(VI) on hydrogel beads. The influence of the organoclay concentration on the OBent (wt.%)/Alg hydrogel beads (1, 2, 3, and 4 wt.%) and the pH (3–8) was studied. Experiments were conducted in glass flasks containing 50 mL of a 25 mg/L aqueous Cr(VI) solution at a controlled pH, to which a specified amount of hydrogel beads were added. Solutions of 0.1 M NaOH and 0.1 M HCl were used to adjust the pH of the solutions. All experiments were conducted under ambient conditions (20 ± 1 °C), with a stirring speed maintained at 150 rpm. The adsorbent was removed by centrifugation at 4000 rpm for 5 min to quantify chromium in the aqueous medium. The supernatant was then filtered through a 0.45 μm membrane, and the chromium concentrations were measured by flame atomic absorption spectrometry (iCE 3500, Thermo Scientific, Waltham, MA, USA), with an air–acetylene flame. All tests were conducted in triplicate. The Cr(VI) removal efficiency was calculated using Equation (2):

(2) $$Cr(VI)\;removal\;\left(\%\right)=\frac{C_{0}-C_{t}}{C_{0}}\times 100$$

where $C_{0}$ and $C_{t}$ represent the initial and remaining Cr(VI) concentrations at a given time (*t*), respectively. The 1,5-diphenylcarbazide colorimetry method was also used for Cr(VI) quantification, following the guidelines of the American Public Health Association (APHA) procedures [67].

### 4.4. Fixed-Bed Column Adsorption Tests

Adsorption tests for (Cr(VI) were conducted using a fixed bed within a glass column that was 20 cm long and 1.0 cm in diameter. This column was filled with a specific quantity of OBent/Alg hydrogel beads. A peristaltic pump (Thomas Scientific 3385, Swedesboro, NJ, USA), equipped with a silicone hose, was connected to the base of the column to deliver the Cr(VI) solution. To ensure consistent flow rates at the column’s inlet and outlet, 1.0 cm diameter glass beads were placed at each end of the hydrogel bed. Before each experiment, water was passed through the column to remove any air bubbles trapped in the fixed bed. All experiments were conducted under ambient conditions (20 ± 1 °C), with the initial pH of the Cr(VI) solution adjusted to a predetermined value. Samples were collected from the column’s top at specified time intervals (until bed saturation), and the Cr(VI) concentration was measured using flame atomic absorption spectrometry and the 1,5-diphenylcarbazide colorimetric method.

The parameters studied in the adsorption of Cr(VI) in a fixed bed were the influence of the feed flow rate (Q = 1, 2, and 3 mL/min), bed height (h = 10, 15, and 20 cm of hydrogel beads), and initial Cr(VI) concentration in the feed ($C_{0}$ = 10, 20, and 30 mg/L). The breakthrough curves were generated by plotting $C_{t}/C_{0}$ (mg/L) against *t* (min), where $C_{t}$ represents the effluent Cr(VI) concentration, $C_{0}$ denotes the influent Cr(VI) concentration, and *t* denotes the service time.

After processing the corresponding breakthrough curve for every fixed bed adsorption test, the parameters listed in Table 6 were determined.

The desorption of the Cr(VI) oxyanions and the reuse capacity of the adsorbent in the fixed-bed column were also evaluated in this study. The adsorbent (OBent (2%)/Alg hydrogel beads) in the fixed-bed column was regenerated by contact with an aqueous NaOH solution at pH 11. Under alkaline conditions, the desorption of Cr(VI) ions is expected due to electrostatic repulsion between the deprotonated functional groups of the organobentonite and the negatively charged Cr(VI) ions [56]. A 0.001 M NaOH solution was fed to the column (bed height of 20 cm and volumetric flow rate of 3 mL/min) for 3 h to quantify the amount of Cr(VI) desorbed in the effluent collected by flame atomic absorption spectrometry. The column was then washed with deionized water, and a new continuous adsorption cycle was performed. The percentage of desorption of Cr(VI) was estimated by the following equation:

$$D\;\left(\%\right)=\frac{m_{des}}{m_{ads}}\times 100$$

where $m_{des}$ (mg) and $m_{ads}$ (mg) are the amounts of desorbed and adsorbed Cr(VI), respectively.

### 4.5. Sample of Tannery Wastewater

The application of OBent/Alg hydrogel beads for Cr(VI) removal was evaluated for the tannery effluent treatment from a tannery company located in the municipality of Belén (Nariño, Colombia). The tannery wastewater (TWW) integrated sample was taken by following the recommendations established guide for monitoring discharges, surface water, and groundwater in Colombia [71]. The TWW sample was refrigerated and taken to the laboratory to analyze several physical parameters, following the standard methods (SMs) for examining water and wastewater [67]. The analyses performed were pH, conductivity, chemical oxygen demand (COD), sulfates, total chromium, and hexavalent chromium–Cr(VI).

## Figures and Tables

**Table 1:** Adsorption parameters of Cr(VI) on OBent (2%)/Alg hydrogel beads in a fixed-bed column.

Q cm	h cm	m g	$C_{o}$ mg/L	$t_{b}$ min	$t_{e}$ min	$V_{t}$ mL	$q_{total}$ mg	$q_{m}$ mg/g	$W_{total}$ mg	$C_{e}$ mg/L	R %
1	10	4.10	20	8.25	323.50	323.5	2.665	0.649	7.60	15.26	35.06
2	10	4.10	20	2.57	243.92	487.8	2.596	0.633	11.60	18.46	22.38
3	10	4.10	20	1.09	183.36	550.1	2.580	0.629	14.40	21.49	17.92
3	10	4.10	20	1.09	183.036	549.1	2.580	0.629	14.40	21.53	17.92
3	15	5.97	20	1.44	264.94	794.8	4.753	0.796	19.20	18.18	24.75
3	20	8.21	20	2.87	353.69	1061.1	7.827	0.954	26.40	17.50	29.65
3	15	5.97	10	2.05	378.83	1136.5	3.463	0.580	13.80	9.10	25.09
3	15	5.97	20	1.44	264.94	794.8	4.753	0.796	19.20	18.18	24.75
3	15	5.97	30	1.09	221.71	665.1	5.673	0.951	24.30	28.01	23.34

**Table 2:** Model for the analysis of the breakthrough curves [33].

Model—Equation	Parameters
**Fractal-like modified Thomas** $\frac{C_{t}}{C_{o}}=\frac{1}{1+exp\left[K_{T,o}t^{-h_{f}}C_{0}\times \left(\frac{q_{o}m}{QC_{0}}-t\right)\right]}$	$C_{o}$ = influent Cr(VI) concentration, mg/L $C_{t}$ = effluent Cr(VI)e concentration (mg/L) at time *t* t = time, min $K_{T,o}$ = fractal-like Thomas rate constant, $L/min\;{mg}^{\left(1-h_{f}\right)}$ $q_{o}$ = adsorption capacity per unit mass of adsorbent, mg/g Q = volumetric flow rate, mL/min $h_{f}$ = fractal-like component m = adsorbent amount, g

**Table 3:** Parameters of the fractal-like modified Thomas model under different conditions.

Q mL/min	h cm	$C_{o}$ mg/mL	$K_{T,o}$ $L/min\;{mg}^{\left(1-h_{f}\right)}$	$q_{o}$ mg/g	$h_{f}$	$R^{2}$	$Red-\chi^{2}$
1	10	20	1.064 ± 0.251	0.519 ± 0.035	0.128 ± 0.053	0.972	0.0031
2	10	20	3.412 ± 1.447	0.265 ± 0.056	0.374 ± 0.091	0.928	0.0058
3	10	20	13.136 ± 2.629	0.135 ± 0.013	0.562 ± 0.046	0.985	0.0014
3	10	20	13.136 ± 2.659	0.135 ± 0.013	0.562 ± 0.047	0.985	0.0014
3	15	20	2.081 ± 0.822	0.311 ± 0.064	0.286 ± 0.084	0.946	0.0041
3	20	20	1.672 ± 0.333	0.484 ± 0.043	0.251 ± 0.042	0.978	0.0021
3	15	10	2.797 ± 0.707	0.235 ± 0.030	0.233 ± 0.051	0.969	0.0023
3	15	20	2.081 ± 0.822	0.311 ± 0.064	0.286 ± 0.084	0.946	0.0041
3	15	30	1.787 ± 1.075	0.399 ± 0.040	0.371 ± 0.083	0.958	0.0032

**Table 4:** Comparison of the Cr(VI) adsorption capacity of different composites.

Adsorbent	Process	pH	$q_{m}$, mg/g	Ref.
Alginate–montmorillonite/polyaniline nanocomposite	Batch	2.0	29.89	[31]
Bio-polymer beads (crosslinked alginate + gelatin)	Batch	6.0	0.833	[35]
Alginate–goethite beads	Batch	3.0	23.38	[54]
Chitosan–citric acid nanoparticles	Batch	3.0	22.4	[55]
Crosslinked chitosan hydrogel functionalized with citric acid	Fixed-bed	3.0	128	[56]
Polyethylene/agave fiber/chitosan composites	Fixed-bed	4.0	5.67	[57]
Magnetic pine cone composite	Fixed-bed	3.0	5.07–33.08	[58]
Organoclay/alginate hydrogel beads	Fixed-bed	3.4	0.519	This study

**Table 6:** Parameters for analyzing fixed-bed adsorption data [16,68,69,70].

Parameter	Symbol and Units	Formulas
Breakthrough time	$t_{b}$ min	Time required for the effluent’s Cr(VI) concentration to reach 10% of that in the influent ($C_{t}/C_{0}\;$= 0.1)
Exhaustion time	$t_{e}$ min	Time required for the effluent’s Cr(VI) concentration to attain 95% of the influent concentration ($C_{t}/C_{0}$ = 0.95)
Treated effluent volume	$V_{t}$ mL	$V_{t}={Q\times t}_{e}$
		Q = volumetric flow rate (mL/min) $t_{e}$ = exhaustion time (min)
Total column capacity	$q_{total}$ mg	$q_{total}=\frac{Q}{1000}\int_{t=0}^{t=t_{total}}C_{ad}dt$
		$C_{ad}=C_{0}-C_{t}$ $C_{ad}$ = adsorbed Cr(V) concentration $C_{0}$ = influent Cr(VI) concentration (mg/L) $C_{t}$ = effluent Cr(VI) concentration (mg/L) $t_{total}$ = total flow time (min)
Maximum adsorption capacity	$q_{m}$ mg/g	$q_{m}=\frac{q_{total}}{m}$
		$q_{total}$ = total column capacity (mg) *m =* total amount of adsorbent in the column (g)
Total amount of adsorbate that entered the column	$W_{total}$ mg	$W_{total}=\frac{C_{o}\times Q\times t_{total}}{1000}$
		$C_{0}$ = influent Cr(VI) concentration (mg/L) Q = volumetric flow rate (mL/min) $t_{total}$ = total flow time (min)
Adsorbate concentration at equilibrium	$C_{e}$ mg/L	$C_{e}=\frac{W_{total}-q_{total}}{V_{t}}$
		$W_{total}$ = total amount of Cr(VI) that entered the column $q_{total}$ = total column capacity (mg)
Total amount of adsorbate removed in the column	R %	$R\left(\%\right)=\frac{q_{total}}{W_{total}}\times 100$
		$q_{total}$ = total column capacity (mg) $W_{total}$ = total amount of Cr(VI) that entered the column

## Data Availability

The original contributions presented in the study are included in the article. Further inquiries can be directed to the corresponding author.
